# Treating Primary Arthroprosthesis Infection Caused by *Mycobacterium abscessus* subsp. a*bscessus*

**DOI:** 10.1155/2019/5892913

**Published:** 2019-12-14

**Authors:** Valerio Pace, Pierluigi Antinolfi, Emanuele Borroni, Daniela Maria Cirillo, Elio Cenci, Claudio Piersimoni, Angela Cardaccia, Marco Nofri, Chiara Papalini, Rosario Petruccelli, Fabrizio Marzano, Maria Bruna Pasticci

**Affiliations:** ^1^Traumatology and Orthopedic Clinic, Department of Surgical Sciences, University of Perugia, Perugia, Italy; ^2^Emerging Bacterial Pathogens Unit, Division of Immunology, Transplantation and Infectious Diseases, IRCCS San Raffaele Scientific Institute, Milan, Italy; ^3^Microbiology Section, Department of Medicine, University of Perugia, Perugia, Italy; ^4^Regional Reference Mycobacteriology Unit-Clinical Pathology Laboratory, United Hospitals, Ancona, Italy; ^5^Infectious Disease Clinic, Department of Medicine, University of Perugia, Perugia, Italy

## Abstract

Prosthetic joint infections (PJI) caused by nontuberculous mycobacteria are very rare, and results of treatment can be unpredictable. A 72-year-old female underwent hip replacement after an accidental fall in a local hospital in Santo Domingo. The postoperative period was uneventful except for a traumatic wound near the surgical scar. PJI caused by *Mycobacterium abscessu*s subsp. *abscessus* was diagnosed 6 months later. A two-stage reimplantation was performed after a 3-month period of aetiology-directed therapy, including amikacin, imipenem, and clarithromycin. *M*. *abscessus* isolate was reported to be resistant to clarithromycin when incubation was protracted for 14 days and to harbour the gene *erm*(41). The patient manifested major side effects to tigecycline. At reimplant, microbiologic investigations resulted negative. Overall, medical treatment was continued for a 7-month period. When discontinued and at 6-month follow-up, the patient was clinically well, inflammatory markers were normal, and the radiography showed well-positioned prosthesis. *Mycobacterium abscessus* subsp. *abscessus* is a very rare cause of PJI, yet it must be included in the differential diagnosis, especially when routine bacteria cultures are reported being negative. Further investigations are needed to determine any correlations between clinical results and *in vitro* susceptibility tests, as well as the clinical implications of *M*. *abscessus* subsp. *abscessus* harbouring the functional gene *erm*(41). Moreover, investigations are needed for determine optimal timings of surgery and lengths of medical therapy to improve patient outcome.

## 1. Introduction

Joint replacements are becoming more common surgical procedures, able to significantly improve the patient quality of life [1, 2]. Minor and major complications have relatively low rates, with reported prosthetic joint infection (PJI) rate of around 1% [3–6]. Infection onset can be early after the surgical procedure or delayed. Risk factors for infection do not significantly vary from those of other types of infections, with the relevant adjunct that revision arthroplasty or history for PJI can particularly increase the risk [6]. Staphylococci and other Gram-positive bacteria are the most common microorganisms isolated from prosthetic joint infection (about 60%). Infections can be also caused by Gram negatives, anaerobes, and fungi. A similar amount of cases have been reported to be polymicrobial or culture negative. *Mycobacterium tuberculosis* and other nontuberculous mycobacteria (NTM) rarely cause PJI [3–6].

The correct aetiology identification of the infection is essential, in order to provide an accurate and adequate treatment [3–5]. Bacterial cultures of synovial fluids, removed prostheses, and intraoperative tissue samplings are recommended before the prescription of any antibiotic therapy [3–5]. With regards to mycobacterial aetiology, routine cultures can lead to false-negative results. Moreover, infections caused by mycobacteria, including *M. tuberculosis* and NTM, are rare and results of treatment can be unpredictable [7–15]. There is a paucity of results in the literature on the treatments and outcomes of mycobacterial PJI [16]; likewise, there are a limited number of publications on PJI caused by *M*. *abscessus* complex. Over the last two decades, the literature has reported the presence of inducible resistance genes for macrolides, which are considered important components in the treatment of infections caused by these microorganisms [17, 18].

We present a case of *M. abscessus* subsp. *abscessus* primary prosthetic hip infection diagnosed and treated.

## 2. Case Report

A 72-year-old female patient presented on the 15^th^ of January 2018 to a local hospital in S. Domingo, after a fall which caused a fracture of her right femur. Three days after admission, the patient underwent a total hip replacement (THR) (posterolateral approach). Clinical findings, routine postoperative blood results, and postoperative radiography controls were all satisfactory, with no signs of complications. Early mobilization and rehabilitation was started on the 1^st^ postop day. The patient was discharged on the 5^th^ postop day and was able to mobilize with two crutches. Routine post-THR instructions were followed by the patient with good compliance. Two weeks later, the patient reported a traumatic superficial wound on the surgical scar that healed spontaneously in a few days. The patient did not refrain from bathing or swimming in the ocean.

The patient returned to Italy on the 11^th^ of March 2018 and continued with the postoperative physiotherapy. However, she started to complain of right inguinal pain, which became progressively worse. A month later, the patient was evaluated at an emergency room because of a red swelling over the proximal part of the wound. The area was drained, and levofloxacin 500 mg a day oral for one week was prescribed. At the next control, no findings were detected.

However, after another month, due to worsening of the pain, the patient was evaluated at the trauma-orthopaedic (TO) clinic of our hospital: Santa Maria della Misericordia, Perugia, Italy. On clinical examination, the range of motion was satisfactory with regards to rotations and extension and hip flexion was limited by pain, but the wound was healed. Radiograph results showed no signs of infection, prosthesis loosening, or other abnormalities.

**Figure 1:**
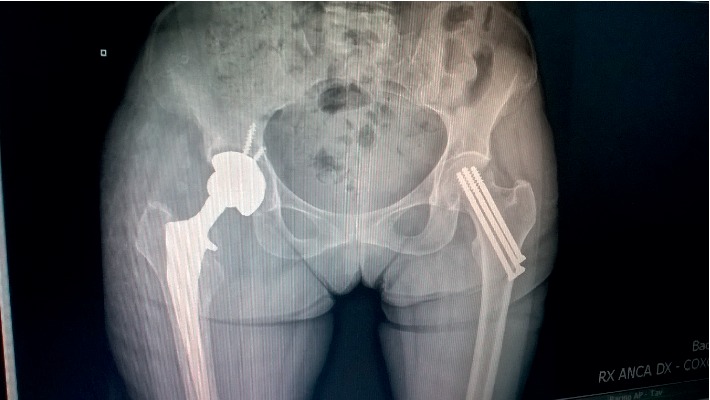
Hip radiography: radiolucency around the acetabular component of the prosthesis.

On the 24^th^ of June 2018, 5 months after surgery, due to ongoing pain and limited hip flexion, the patient underwent a repeated radiography. At this time, a radiolucency around the acetabular component of the prosthesis was evidenced (Figure 1). An ultrasonography of the right hip showed multiple little fluid areas around the prosthesis with the aspect of multiple abscesses. Therefore, the patient was admitted to the TO clinic.

On admission (29^th^ of June 2018), past medical history included hypercholesterolemia, previous atrial fibrillation (not currently in treatment), gastritis, and aortic valve replacement in July 2017 (biologic prosthesis). She denied any allergy. Drug history included proton pump inhibitors, escitalopram, simvastatin, and pain killers when required. She denied smoking and drinking. The physical examination evidenced normal vital signs, a systolic murmur, and a limited motion of the hip due to pain. Only the day of admission temperature 37.8°C was recorded. Laboratory investigations showed erythrocyte sedimentation rate (ESR) 85 (normal range: 1–30 1^st^ hour), C-reactive protein (CRP) 5.6 mg/dL (normal range 0.0–0.5 mg/dL), and white blood count (WBC) 5.34 (normal range: 3.60–9.60 × 10^3^/*µ*L). Liver and kidney function tests were normal. An ultrasound-guided right hip joint aspirate was performed on the 2^nd^ of July and sent for microbiological investigations. Also urine and blood cultures were collected and both resulted negative. Both transthoracic and a transesophageal echocardiography were performed, and both showed the absence of cardiac valve vegetations or any other relevant cardiac abnormality. The biologic valve, which had been replaced in 2017, was well functioning. Teicoplanin 400 mg/day IV and imipenem 500 mg TID IV were started on the 2^nd^ of July (patient's weight was 45 kg).

However, the periprosthetic fluid aspirate (1 sample) results on the 11^th^ of July showed growth of acid-fast bacilli (AFB), identified as *Mycobacterium abscessus*. Given these findings, a further aspirate was obtained on the same day to confirm the result. The sample was examined also with acid-fast stain, which resulted negative for AFB (Table 1). Teicoplanin was discontinued while imipenem was confirmed, and clarithromycin 500 mg BID orally and amikacin 500 mg once a day IV were started (Table 1). Meanwhile, a revision procedure was planned and performed the day after. The previous scar and a posterolateral approach were used. No fistulas were noticed, and the state of the skin around the right hip was satisfactory with no redness or palpable masses. However, there was purulence between the bone and acetabular part of the prosthesis, and the acetabular component was loosed. On the contrary, the femoral part of the prosthesis was stable. A femoral fracture occurred while removing the prosthesis. A vancomycin-gentamycin antibiotic-loaded spacer was implanted. An intraoperative bone sample along with the explanted prosthesis was collected for microbiologic studies with the sonication technique. Microscopy examination evidenced purulence but microorganisms were not observed. Culture results were obtained on the 20^th^ of July, and evidenced *M. abscessus* growth (antibiotic susceptibility tests are reported in Table 2) along with *Enterococcus faecalis*. Both microorganisms grew only from the sonication fluid of the prosthesis broth culture. *E*. *faecalis* was susceptible to ampicillin, teicoplanin, vancomycin, and tigecycline. The ongoing antibiotic treatment was maintained, and linezolid 600 mg BID orally was added (Table 1).

**Figure 2:**
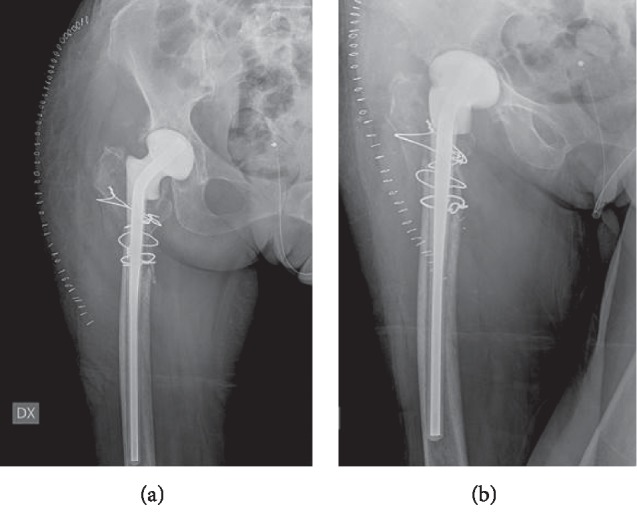
Hip radiography: anteroposterior and oblique views after prosthesis explant and spacer positioning.

The postoperative radiography showed satisfactory results (Figure 2).

A week later, a PICC-line catheter was inserted, and after 4 days, the patient was transferred initially to the Infectious Disease Clinic of our hospital and then to a rehabilitation centre where the patient continued on the antibiotic treatment and clinical and laboratory monitoring (Table 1).

Drug susceptibility testing (DST), was performed on the *M. abscessus* subsp. *abscessus* isolate using the *E*-test (AB Biodisk, Solna, Sweden) and the broth microdilution methods, the former with both 4 day and 14 day readings (Sensititre, Trek Diagnostic Systems, USA) [20, 21]. Results were evaluated according to the breakpoints reported in the CLSI document from 2011 (Table 2) [19].

In addition to phenotypic DST, the isolate also underwent gene sequencing using the Illumina platform MiniSeq (Table 2). Results revealed the presence of the wild-type gene *erm*(41) conferring inducible clarithromycin resistance [17, 20, 22, 23], while no mutations for the gene 23s affecting macrolide susceptibility and the gene 16s affecting amikacin susceptibility were found.

The patient was readmitted on the 20^th^ of October, 3 months after the prosthesis had been explanted, at the Infectious Disease Clinic to be evaluated for prosthesis reimplant. On the day of admission, amikacin was discontinued (Table 1).

On admission, the patient was clinically and hemodynamically well, wound and surrounding skin conditions did not show findings of infection, and range of motion was limited as expected for the presence of the spacer. Laboratory examinations were in the normal range.

Due to the *in vitro* susceptibility tests and gene sequencing results, antibiotic treatment was modified (Table 1). A two stage revision procedure was performed on the 14^th^ of November, and a new hip prosthesis was implanted. Again, the previous scar and the posterolateral approach were used. The spacer was removed and sent together with a bone sample to the microbiology laboratory for sonication cultures that resulted negative for *M*. *abscessus* and any other microorganism. Urine and blood cultures were also drawn and were negative.

**Figure 3:**
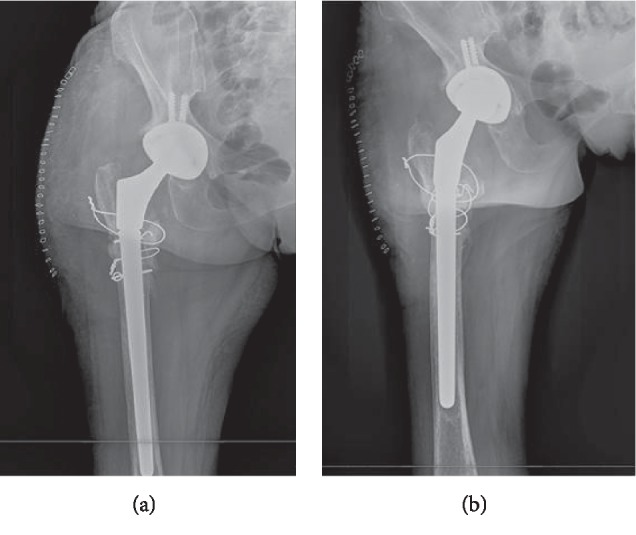
Hip radiography after prosthesis reimplant.

In the postoperative period, antibiotic treatment was further modified due to tigecycline intolerance (Table 1). Thereafter, the patient was clinically well, without signs of local or systemic infection. Postoperative radiography showed good position of the prosthesis (Figure 3).

At discharge, pain was under control, skin and wound in good conditions, the mobility status was satisfactory, and antimicrobial therapy consisted of amikacin 500 mg IV once a day, clarithromycin 500 mg orally BID, and imipenem 500 mg TID IV (Table 1).

The follow-up every 2 weeks included clinical evaluation and inflammatory markers as well as liver and renal function tests (Table 1).

In December 2018, the patient was admitted because laboratory parameter evidenced an increase of ESR and PCR values. On admission, the patient was feeling well, she had no fever, and on physical examination there were no systemic or local signs of infection over the right hip. The PICC-line was changed, and cultures resulted negative. ESR resulted normal five days later. *M*. *abscessus* directed therapy was again modified discontinuing imipenem and amikacin and adding tigecycline 50 mg twice a day IV to clarythromicin. Eight days later, tigecycline had to be interrupted again due to gastric intolerance and increased transaminases.

Eighteen weeks after prosthesis was reimplanted, the patient had no pain, the prosthesis was functioning well, the inflammatory parameters were normal, and clarithromycin, the only antibiotic left for the last 3 weeks, was discontinued (Table 1).

At follow-up 6 months after treatment discontinuation, the patient had no pain, inflammatory markers were normal, and prosthesis was functioning well.

## 3. Discussion

We present a case of PJI caused by *Mycobacterium abscessus* subsp. *abscessus*.

*Mycobacterium abscessus* subsp. *abscessus* PJI was defined by the presence of clinical manifestations and the growth of the microorganism from the periprosthetic aspirate and the sonication fluid of the prosthesis.

*M. abscessus* subsp. *abscessus* belongs to rapidly growing NTM. Its growth requires specific media, yet it can develop also into routine bacterial plates or broth culture media in about one week. However, its identification can be missed when cultures are incubated for only 48 hours [10, 14, 16, 24].

Overall, infections due to member species of the *M*. *abscessus* complex are not uncommon and are consequent to the exposition to these microorganisms. Nevertheless, the association between exposition and infection is difficult to diagnose and usually reported only for health care-associated infections or outbreaks following specific procedures. *M*. *abscessus* complex most commonly causes skin, soft tissue, and pulmonary infections; however, any human tissue can be involved. The complex rarely has been reported to cause PJI, and usually it is in the early postoperative period [10–16, 24]. In our patient, clinical symptoms started within 3 months from surgery, and it is not clear if the prosthesis infection occurred when it was first implanted or afterwards, when the patient reported a traumatic skin wound and eventually tissue exposition to mycobacteria present in tap water or in the environment.

Symptoms of PJI caused by nontuberculous mycobacteria are nonspecific. In our patient, the only symptom was pain that started soon after the prosthesis was implanted. At that time, there were no indications for further investigation because pain can be a common finding in most cases of joint arthroplasty in the early postoperative period and it is usually related to the procedure itself. As a result, the first time we had a suspicion of infection was 6 months after surgery when the radiography evidenced a radiolucency around the acetabular component of the prosthesis, and above all, when the ultrasonography showed multiple abscesses surrounding the prosthesis, the inflammatory markers resulted to be elevated and *M*. *abscessus* was isolated from the synovial fluid aspirate. Overall, the clinical course was that of a subacute, early/delayed PJI.

With regard to treatment, the past studies have reported correlations between treatment protocols and outcomes for NTM prosthetic joint infections. Nevertheless, there are no treatment protocols relating to *in vitro* susceptibility tests, gene studies, and the clinical response, especially when *M*. *abscessus* PJI is diagnosed [10–16, 23–25]. Overall, in order to treat the patient and prevent recurrence, there is an agreement that a combination of antibiotics and a long-course therapy are best indicated. Among the antibiotics, amikacin is reported to lead to good outcome, together with clarithromycin, fluoroquinolones, and imipenem [10–16, 23–25]. However, *M. abscessus* is a complex and comprises three different subspecies: *M. abscessus* subsp. *abscessus*, *M. abscessus* subsp. *bolletii*, and *M. abscessus* subsp. *massiliense* [23, 26] that may have different susceptibility patterns. Specifically, *M. abscessus* subsp. *abscessus* and *M. bolletii* might harbour a functional gene *erm*(41) leading to lack of efficacy of macrolide; therein, raising questions on the utility of clarithromycin in the treatment of infections caused by these microorganisms with inducible macrolide resistance [18, 27, 28]. To investigate for this probability, gene sequencing is advisable. However, when this is not possible *in vitro* susceptibility tests with also an extended incubation periods are recommended [19, 22, 29].

As in the majority of patients with PJI, while awaiting synovial fluid aspirate results, the initial treatment for our patient was empiric, directed against the more common microorganisms causing PJI. The identification of *M*. *abscessus* as the aetiology of PJI in our patient necessitated the resetting of the therapeutic regime, following indications from the current literature and the *E*-test susceptibility reports with shorter incubation time. According to those results, imipenem, clarithromycin, and amikacin were active against the isolate [19]. However, contradictory results were obtained when clarithromycin activity was evaluated with broth microdilition technique and extending the incubation period to 14 days [19]. The same issue emerged also for imipenem. Being so, while a prolonged incubation is recommended to test clarithromycin *in vitro* activity [19, 29, 30], a long incubation time can cause the inactivation of other antibiotics such as imipenem [28, 30]. In light of this, the reported results might have been misleading. Overall, these *in vitro* tests were time consuming and caused delays and doubts on the efficacy of the antibiotics we were administering. In treating our patient, we were also faced with a delay in the isolate identification at the subspecies level, as well as the results of the genetic investigations evaluating for the presence of the inducible gene *erm*(41). The presence of this gene has been reported associated with clarithromycin resistance. The patient also encountered side effect experienced from the tigecycline administration, which was reported to have a good *in vitro* activity against the patient *M*. *abscessus* isolate.

In our patient, *E*. *faecalis* was also grown from the sonication fluid of the prosthesis, but it was diagnosed as a contamination, as it grew only from one sample and only in the broth culture. Nevertheless, even if *E*. *faecalis* had an aetiological significance, it was ampicillin susceptible, therefore, susceptible to imipenem, and the patient was treated with imipenem for 5 months.

According to scientific evidence, debridement and implant removal are both very important in the treatment of PJI infections, including those caused by NTM. Most favourable outcomes seem to be related to a two-stage revision surgery strategy with removal of the infected prosthesis and insertion of the antibiotic cement spacer (1^st^ stage) to be followed by timely reimplantation of a new prosthesis. We followed the aforementioned recommendations [10–16]. The medical treatment in this case consisted of amikacin, imipenem, and clarithromycin, for 7 months and, overall, this combination of antibiotics in addition to surgery revealed to be successful.

With regard to clinical and functional outcomes, the patient exhibited a good range of motion before discharge and at an early (6 months) follow-up was pain free and able to walk. The wound was well healed with no evidence of local infection. The patient also referred to be satisfied with the treatments, and regained her quality of life.

## 4. Conclusions

*Mycobacterium abscessus* subsp. *abscessus* is a very rare cause of PJI, yet it must be included in the differential diagnosis, especially when routine bacteria cultures are reported being negative. Further investigations are needed to determine any correlations between clinical results and *in vitro* susceptibility tests, as well as the clinical implications in treating infections caused by *M. abscessus* subsp. *abscessus* harbouring the functional gene *erm*(41). Finally, investigations are needed to study optimal timings of surgery and lengths of medical therapy.

## Figures and Tables

**Table 1:** Microbiology, medical, and surgical treatments.

Date	Microbiology results	Medical therapy	Surgical procedures	Side effects
07/02/2018	Periprosthesis fluid: *M. abscessus*	Imipenem, teicoplanin	Periprosthetic aspirate	

07/11	Periprosthesis fluid: negative	Imipenem, amikacin, clarithromycin	Periprosthetic aspirate	

07/12	Prosthesis: *M*. *abscessus* + *E*. *faecalis* Periprosthesis bone: negative	Imipenem, amikacin, clarithromycin, linezolid	Prosthesis explanted vancomycin + gentamycin-loaded spacer	

07/27		Imipenem, amikacin, clarithromycin	PICC-line positioned	

08/09		Imipenem, amikacin, clarithromycin		

08/17		Imipenem, amikacin, clarithromycin		

09/18		Imipenem, amikacin, clarithromycin		

10/20		Imipenem, clarithromycin		Suspected amikacin ototoxicity

10/26		Tigecycline, amikacin		Probable *in vitro* imipenem resistance

11/10		Imipenem, amikacin		Tigecycline gastric intolerance

11/14	Spacer + bone samples: no growth	Imipenem, amikacin,	Prosthesis reimplant	

11/27		Imipenem, amikacin, clarithromycin		

12/04		Imipenem, amikacin, clarithromycin		

12/05		Imipenem, clarithromycin	PICC-line explanted	Suspected amikacin ototoxicity

12/10		Imipenem, clarithromycin	PICC-line positioned	

12/12		Tigecycline, clarithromycin		Tigecycline gastric intolerance and hepatitis

12/20		Clarithromycin		

03/01/2019		Clarithromycin	PICC-line explanted	Stop clarithromycin

**Table 2:** *Mycobacterium abscessus* subsp. *abscessus*: *in vitro* antibiotic susceptibility and genome sequencing results. Breakpoints according to CLSI [19].

Antibiotics	07.02.2018	08.07.2018		10.01.2018	10.02.2018
	^*∗*^4-day reading (mg/L)	^*∗*^4-day reading (mg/L)	^*∗*^14-day reading (mg/L)	^*∗∗*^14-day reading (mg/L)	
					23s-wild type-macrolide constitutive resistance not detected
					16s-wild type-susceptible to amikacin *erm*(41)-wild type-macrolide-inducible resistance detected
Amikacin	3 (S)			16 (S)	
Tigecyclin	0.5 (S)			0.75 (S)	
Clarithromycin	0.5 (S)	1.5 (S)	4 (R)	4 (R)	
Imipenem	4 (S)			32 (R)	
Linezolid	8 (R)			>256 (R)	
Azitromycin	128 (R)				
Moxifloxacin	>32 (R)				
Gentamycin	12 (R)				

^*∗*^ *E*-test, Middlebrook 7H-11 agar; ^*∗∗*^broth microdilution testing, Middlebrook 7H-9 broth.
